# GMO analysis results from official food control laboratories in Germany from 2017 to 2021

**DOI:** 10.1007/s00003-023-01425-0

**Published:** 2023-03-06

**Authors:** Hans-Ulrich Waiblinger, Christine A. Eichner, Gabriele Näumann, Ulrich Busch

**Affiliations:** 1 State Institute for Chemical and Veterinary Analysis Freiburg (CVUA Freiburg), Freiburg, Germany; 2 Lower Saxony State Office for Consumer Protection and Food Safety, Food and Veterinary Institute Braunschweig, Hannover, Germany; 3 Institute for Hygiene and Environment, Hamburg, Germany; 4 Bavarian Health and Food Safety Authority (LGL), Oberschleißheim, Erlangen, Germany

**Keywords:** GMO analysis, Genetically modified food, Agriculture, Food control, Official control laboratories

## Abstract

In Germany, genetically modified organisms (GMO) analysis of food samples collected within the official food control is performed by the laboratories of the Federal States. The present report shows GMO analysis results from food samples of the years 2017 to 2021, including contaminations by unauthorized GMO, as well as genetically modified (GM) plant events authorized in the European Union. In addition to previous publications, evaluation of the aggregated food samples analysed for GMO components is shown. During this timeframe, 1077 (7.1%) out of 15,145 samples contained genetic modification. In 43 samples, DNA sequences of unauthorized GM plants were found. Additionally, for food derived from soybean, evaluations according to different product categories and the agronomic production (conventional and organic farming) are shown. Whereas in products from organic farming and in conventional soybeans labelled “without genetic engineering” GM soybeans were detected in 6.1% and 8.9%, of all tested samples, respectively, nearly 30% of all conventional soy samples yielded positive results below 0.1%. However, only in 0.7% of the overall analysed 5424 soybean samples GMO percentages of more than 0.1% were obtained. Generally, authorized GM plants were only found at low contamination levels. The labelling threshold of 0.9% for GM ingredients was exceeded only in 0.2% (maize) and 0.1% (soybean) samples, respectively. For monitoring purposes and risk evaluation, the data collection shall be continued.

## Introduction

One of the major responsibilities of food control authorities is food monitoring for genetic modifications. In Europe, the Regulations (EC) No. 1829/2003 and 1830/2003 define the specific requirements for labelling and authorization of GMO and products thereof in food and feed. In Germany, the Federal States are responsible for food control. Food is analysed for the presence of GM components, and the results are summarized and evaluated in laboratories of the Federal States (ALS 2022). This practise has been performed for more than 15 years by these laboratories. Originally, data collection was performed based on the interpretation of individual results and the labelling requirements defined in the Regulations (EC) No. 1829/2003 and (EC) No. 1830/2003. And according to these regulations, GMO labelling was not necessary if a positive GMO result was below the labelling threshold of 0.9%, provided that this GMO proportion was “technically unavoidable” or “adventitious”. This simple and pragmatic approach compromised quality control measures and documentation, and presented findings with comparable products. In previous years, the results of the German official food control were obtained and published based on the above mentioned practise (Waiblinger et al. 2007, 2011, 2014, 2018). Following this approach, the results of GMO analysis of the German official food control laboratories from 2017 to 2021 are presented and discussed in this paper. In addition to previous publications, evaluation of the aggregated food samples analysed for GMO components is shown.

## Current situation

Worldwide, genetically modified (GM) plants are commercially cultivated in 29 countries on more than 190 million hectares. The top five producing countries of GM crops are the USA, Brazil, Argentina, Canada, and India (ISAAA 2023).

Various genetically modified lines (events) of important crops, such as maize, soybean and canola, are commercially grown on large acreages, especially in North and South America. In 2019, on 74% and 31% of the entire acreages GM soybeans and maize were cultivated, respectively (ISAAA 2023). In Argentina, the average biotech crop adoption rate is nearly 100% (saturation) (ISAAA 2023). Even in Brazil, which is considered as the most important country for cultivation of non-GM soybeans, the amount of non-GM soya is reducing. Consequently, the cultivation of GM soy was increased up to 95%, which is close to saturation in 2019 (Transgen 2023).

In contrast to the North- and South American countries, the cultivation of GM crops in Europe is continuously decreasing. In 2022 only on around 1% of the whole acreage GM crops were cultivated (Transgen 2023). In the EU, the number of deliberate releases of GM plants has been reduced to the cultivation of *Bacillus thuringiensis* (Bt)-maize in Spain and Portugal. In Spain, only 21.3% of the overall maize production is GM maize (Transgen 2023), suggesting that the cultivation of GM crops will not play an important role in the EU in the near future.

By contrast, the number of GM plants in the EU authorized for food purposes is increasing. Here, the authorities cover the import of the GM plants from countries where they are cultivated, as well as the processing of these plants. In December 2022, 41 authorizations of events and stacked events of maize, 26 of soybeans, 7 of canola, 1 of sugar beet, and 15 of cotton were listed in the EU register (EC: EU Register of authorized GMOs 2023).

## Overall GMO analysis

**Fig. 1:**
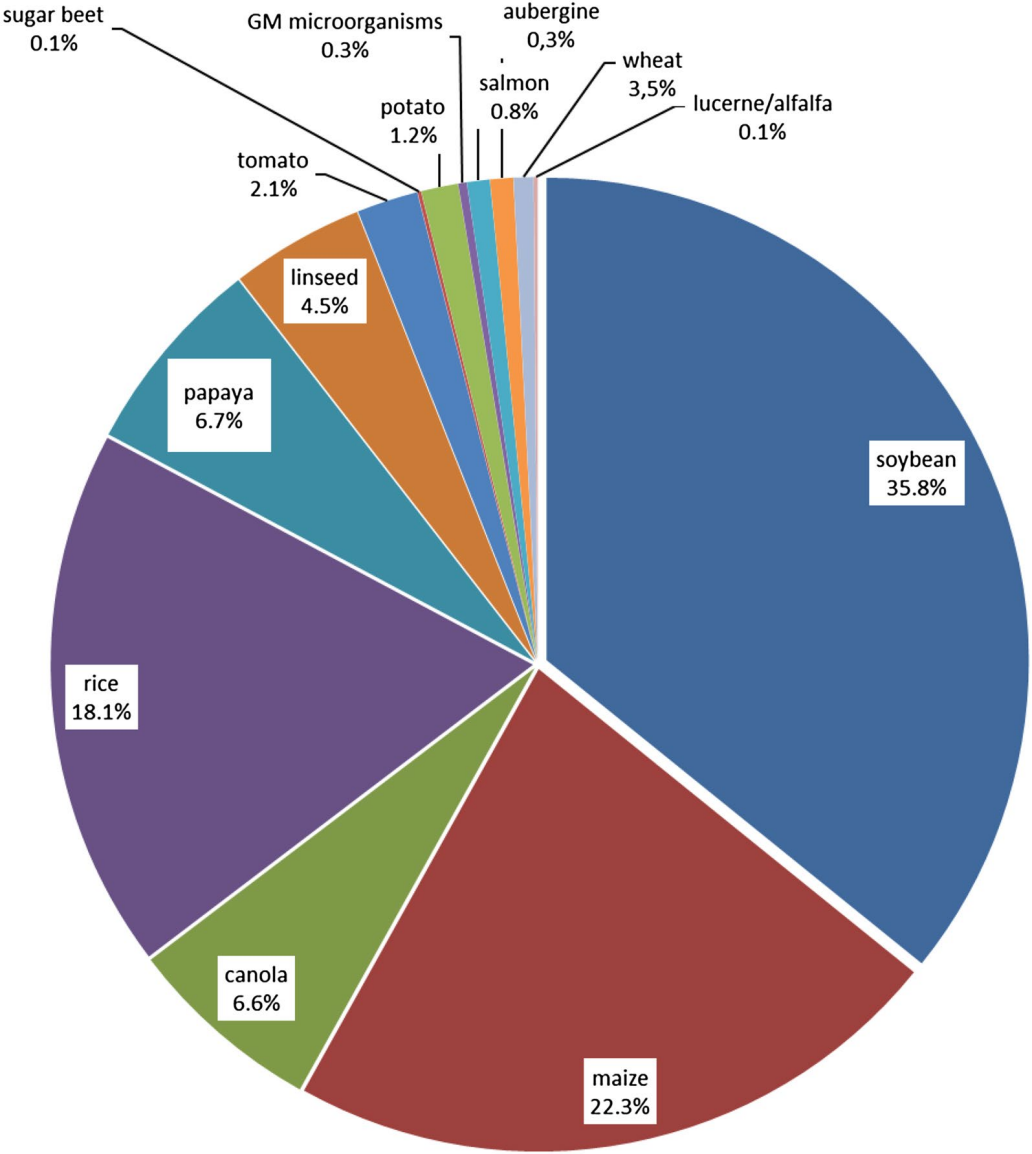
Food samples analysed for the presence of genetic modifications by official German control laboratories in the years 2017 to 2021 [percentage per species, n (total) = 15,145 samples]

The overall numbers of food samples analysed for GMO constituents by the German official control laboratories in the years 2017 to 2021 are shown in Fig. 1, specified for each species.

**Fig. 2:**
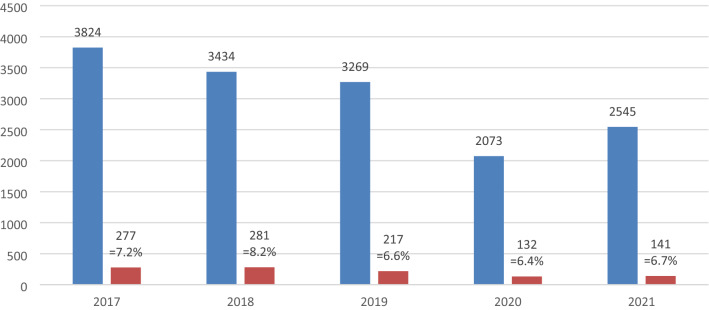
Food samples analysed for the presence of genetic modifications by official German control laboratories from 2017 to 2021 [total number of samples (blue bars) and number of GMO positives thereof (red bars)]

In this study, mainly food samples containing soybean, maize, rice and canola were analysed. A considerable sample number was also tested for the presence of GM papaya, linseed, tomato and wheat. Other species, including potato, salmon, alfalfa, sugar beet or aubergine were only analysed sporadically. Figure 2 illustrates the samples for the GMO analysis per year. 1077 (7.1%) of 15,145 samples were considered positive. The proportion of positive results for GMO analysis stayed constant during the sampling period, in the range between 6 and 8%.

**Fig. 3:**
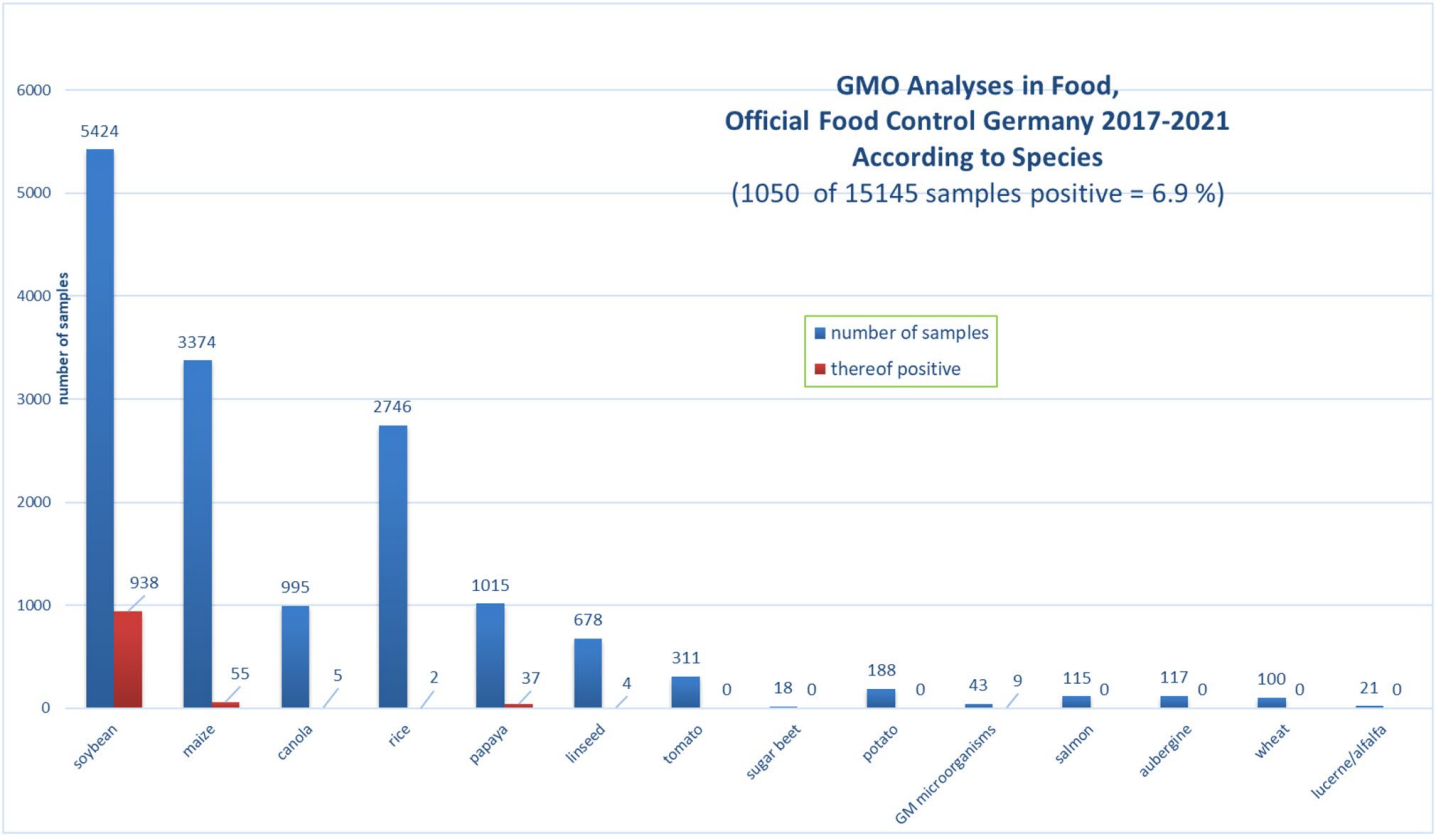
Food samples analysed for the presence of genetic modifications by official German control laboratories from 2017 to 2021 [total number of samples per species and number of GMO positives thereof]

The lower sample numbers between 2020 and 2021 were due to the COVID-19 pandemic. Restricted access of inspectors to premises, as well as additional duties of the laboratories in COVID-19 analysis are considered as main reasons for the reduced number of samples. More specifically, the ratio of positive samples per species is shown in Fig. 3.

Figure 3 illustrates that 17.3% of food samples containing ingredients derived from soybean were analysed positive for GM, indicating the highest percentage obtained among all tested species. The positive results were followed by foods with papaya and maize ingredients with 3.6% and 1.6%, respectively.

## Unauthorized GMO

Components of GM plants that are not authorized within the EU were found in samples of food with papaya, rice and linseed.

In 3.6% of the tested papaya samples (37 out of 1015 samples), DNA sequences from GM papaya were detected. Event 16-0-1 was identified in 4 samples, whereas for the other samples, only construct-specific sequences were identified (mostly the gene construct of the papaya ringspot virus coat protein and the nos Terminator). In 0.6% of the analysed linseed samples (4 out of 678 samples) GM linseed (flax) of the event FP 967 was found. In 2 out of 2746 rice samples, unauthorized GM rice was identified. In both cases, an explicit specification was not possible. Kefeng6 was most probably present in one rice noodle sample; another sample of gluten-free pasta on rice basis contained the DNA constructs of the cauliflower mosaic virus 35 S promoter, the herbicide-tolerance gene bar (P35S/bar), and the *Agrobacterium tumefaciens* nos terminator and the herbicide-tolerance gene bar (Tnos/bar).

Except for canola, soybean and maize (see below), GMO specific DNA was not detected in food derived from other plant species with GMO relevance. The analyses of tomato, potato, sugar beet, wheat, lucerne (alfalfa), aubergine and salmon samples were negative for GM.

## Authorized GMO

### Canola, soybean and maize

Most of the GM positive samples derived from GM soybean, maize and canola ingredients and only originated from GM plants authorized in the EU. In 5 out of 995 canola samples (including mustard with potential botanical impurities from canola) GM canola was detected. These findings could be traced back to low amounts of event GT73.

**Fig. 4:**
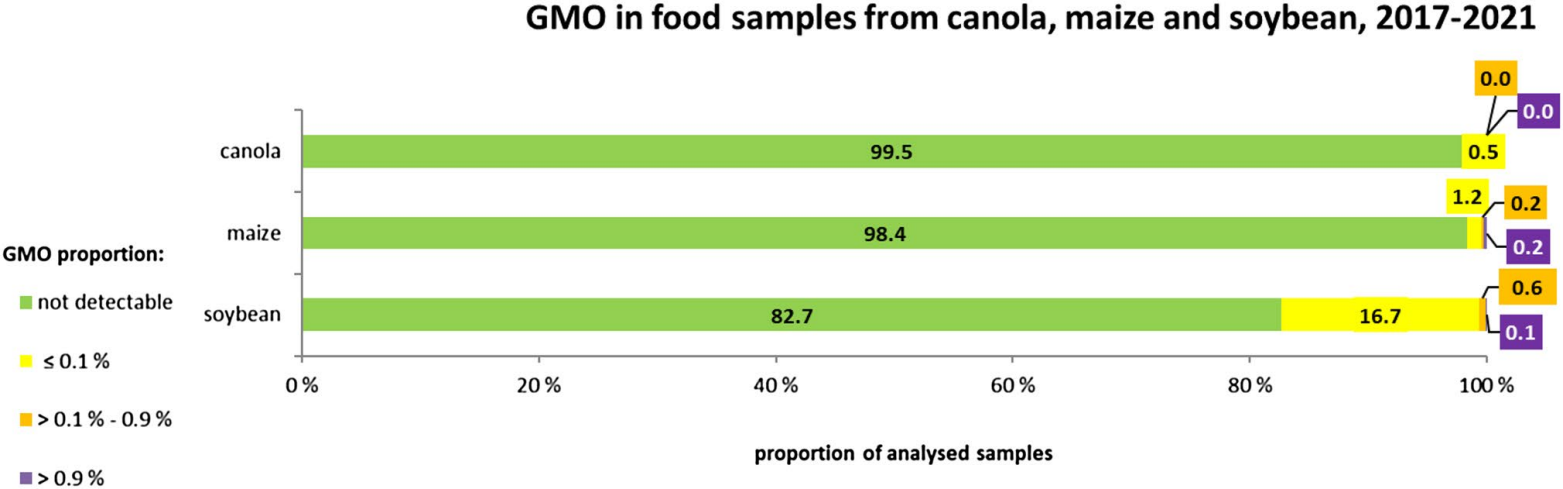
Detection and quantification of GM soy, maize and canola in food, proportions of analysed samples with different levels of GMO percentages. Results from German food control samples between 2017 and 2021

Similar to the rapeseed findings, a low contamination level was observed for soy and maize products (Fig. 4). For maize, 0.4% of the 3374 tested samples exceeded the 0.1% level for reliable quantification of GM maize. For soybean, this level was exceeded in 37 (0.7%) out of 5424 samples. The labelling threshold of 0.9% for GM ingredients was exceeded in 0.2% (maize) and 0.1% (soybean) samples, respectively.

As shown in Fig. 4, differences between samples based on soybean and maize can especially be identified in the proportion of positive samples (17.3% vs. 1.6%). However, most of the positive soybean samples (16.7%) demonstrated a very low level (equal to or below 0.1%) of contamination.

### Detected events

Only DNA sequences derived from GM plants of rapeseed, soybean, and maize authorized in the EU were identified in this study (Table 1).

**Table 1:** Specification of detected events of GM soybeans and maize and their percentage from food samples collected between 2017 to 2021

	GMO percentage and name of the event		
	** ≤ 0.1% > 0.9%**	** > 0.1–0.9%**	** > 0.9%**
Soybean	**A2704-12**	A2704-12	A2704-12
	A5547-127	**GTS 40-3-2**	FG72
	DP305423	MON87708	**GTS 40-3-2**
	**GTS 40-3-2**	**MON89788**	MON87708
	**MON87701**		**MON89788**
	**MON87708**		
	**MON89788**		
Maize	59122	MON 810	59122
	Bt11	MON88017	Bt11
	GA21	MON89034	GA21
	MIR162	NK603	MIR162
	MON810		MIR604
	MON863		NK603
	MON88017		MON810
	MON87460		MON88017
	MON89034		MON89034
	**NK603**		T25
	**TC1507**		TC 1507

In **bold:** event detected at least by 3 Federal States in Germany

### Soybean products in detail

**Fig. 5:**
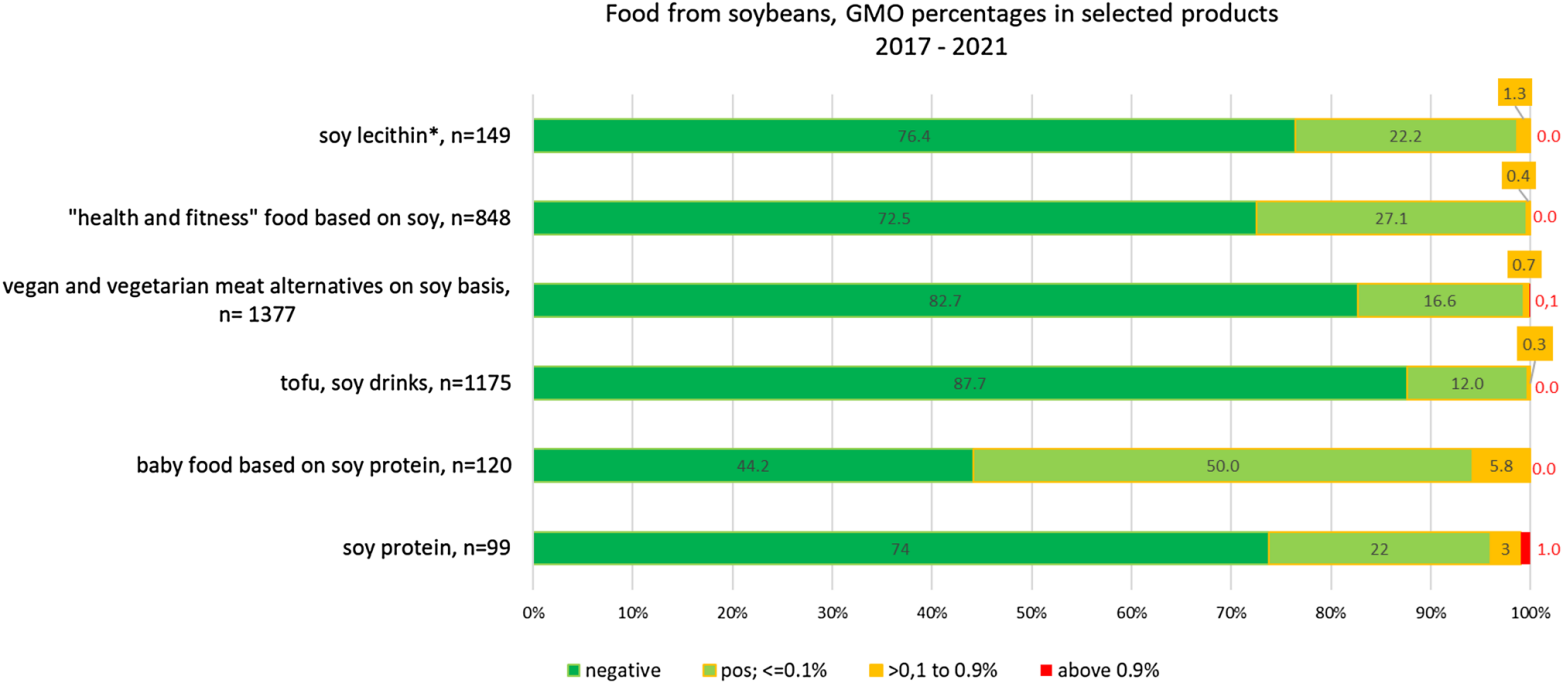
The detection and quantification of GM soy in selected soy products [%]. Results were obtained from German food control samples between 2017 and 2021. n = number of samples. * only samples with sufficient soybean DNA for further analyses (practical limit of detection below 0.9%)

Figure 5 summarizes the results of the GMO analyses in selected soybean products that have been obtained by German official food control laboratories between 2017 and 2021. For the most important categories of soy products, the percentages of GM soybean are described.

In some these food categories, the degree of GM contamination differs. For example, soy protein isolates and soy protein based baby food showed a higher percentage of GM contamination (3.0% and 5.8%, respectively). GM amounts > 0.1% was detected in soy compared to other food categories, including 0.3% for tofu and soy drinks.

### 95th percentile values

In our previous publications (Waiblinger et al. 2007, 2011, 2014, 2018), we presented 95th percentile values, expressed in percent GM soybean or maize and specified in categories of products. Each 95th percentile value represents the GMO percentage not exceeding in 95% of all samples for a given food category.

95th percentile value of more than 0.1% for a food category was calculated lastly in 2017. In soy protein based baby food, this value was 0.22%. Since 2018, 95th percentiles were below 0.1% for all food categories, including protein based baby food. Before 2011, considerably higher 95th percentiles were obtained in some food categories (e.g., up to 0.5% for soy based sports nutrition products (Waiblinger et al. 2007, 2011, 2014).

The German official food control uses the 95th percentile to evaluate if a contamination by GM plant ingredients is “technically unavoidable” or not (Waiblinger et al. 2007, 2011, 2014, 2018). Based on this, in 2017 the availability of GM in baby food, including soy protein isolates with a contamination of 0.22% GM soybean (or less) was feasible on the market.

The food industry produces more and more in countries without any (official) cultivation of GM plants, avoiding countries with coexistence of GM and non GM plants. This might be an explanation for the decrease of the 95th percentiles in all soy products categories in the considered period.

For food control practice, the current values imply that even GMO percentages of 0.2% can be labelled as “technically unavoidable” in alignment with regulation (EC) 1829/2003.

### GM soybean: organic vs. conventional cultivation

**Fig. 6:**
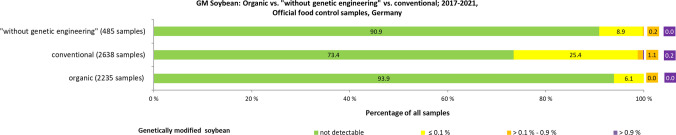
Detection and quantification of GM soy in soy product samples from organic and conventional production. In addition, results for conventional products labelled with “ohne Gentechnik” (= without genetic engineering) are shown

Figure 6 illustrates the overall results for soybean products from conventional and organic farming, sampled between 2017 to 2021. Additionally, results of conventional soy product samples are shown, labelled with “ohne Gentechnik” (= ”without genetic engineering”), legally defined in Germany.

Ingredients from GM plants might not be used for production of organic food, and food products labelled as "without genetic engineering".

The German regulation for products labelled with “without genetic engineering” does not include a labelling threshold (EC Genetic Engineering Implementation Act (2004). If at all, only minimal traces of GMO contaminations below the analytical limit of quantification (< 0.1%) is allowed.

Only little amounts of GM soy in both soybean product categories, including products derived from organic farming, and conventional soybean products labelled with “without genetic engineering” were detected. In samples from organic farming, GM soybean was not detected > 0.1%. Yet, one sample labelled as “without genetic engineering” contained 0.2% GM soybean. However, all results were < 0.9% threshold. Traces of GM soybeans were detected in 6.1% (organic) and 8.9% (“without genetic engineering”) of all tested samples, respectively.

These results clearly differed from those obtained from conventional soy products. Around 27% of all conventional soy samples yielded positive results, and for 1.3% of these samples GMO percentages of more than 0.1% were obtained.

In general, the obtained results are comparable to the ones reported in the years between 2012 and 2016 (Waiblinger et al. 2018).

## GM microorganisms

During the sampling period, 43 samples, mostly enzyme preparations containing amylases, were analysed for the presence of DNA constructs specific of GM microorganisms. In 9 samples, the construct of the pUB110 shuttle vector and a *Bacillus* gene coding for α-amylase (pUB110/α-amylase) was detected. Currently, the legal status of these enzyme preparations in terms of labelling requirements and/or authorization is not clarified, especially if only DNA but no viable GM microorganism can be detected.

## Conclusion

In this work, an overview of the results of all food samples analysed for GM constituents by the official food control laboratories in Germany in the years 2017 to 2021 is given. Whereas the overall percentage of positive samples and the level of contamination is low, the spectrum of the detected GM events is increasing. For GM plants and other GM products not authorized within the EU even small traces are not permitted.

This evaluation shall give assistance to the control laboratories and authorities for risk-based planning of food sampling in the future and shall therefore be continued.
